# KIF11 manipulates SREBP2‐dependent mevalonate cross talk to promote tumor progression in pancreatic ductal adenocarcinoma

**DOI:** 10.1002/cam4.4683

**Published:** 2022-05-26

**Authors:** Xiang Gu, Qunshan Zhu, Guangyu Tian, Wenbo Song, Tao Wang, Ali Wang, Xiaojun Chen, Songbing Qin

**Affiliations:** ^1^ Department of Radiotherapy The First Affiliated Hospital of Soochow University Suzhou China; ^2^ Department of Oncology Jiangdu People's Hospital Affiliated to Medical College of Yangzhou University Yangzhou China; ^3^ Department of General Surgery Jiangdu People's Hospital Affiliated to Medical College of Yangzhou University Yangzhou China

**Keywords:** KIF11, mevalonate metabolism, PDAC, SREBP2, ubiquitination

## Abstract

Cholesterol metabolism is highly correlated with risks of pancreatic ductal adenocarcinoma (PDAC). Nevertheless, the underlying mechanisms of activation of cholesterol biogenesis remain inconclusive. KIF11 is a key component of the bipolar spindle and expresses highly in various malignancies. However, its functional role in PDAC tumorigenesis is still unclear. This study aims to elucidate the oncogenic functions of KIF11 in stimulating cholesterol metabolism, thereby driving PDAC progression. We utilized bioinformatics analysis to identify that KIF11 expressed highly in tumor samples versus paired normal tissues and high KIF11 correlated with high clinical stages of patients. Patients with high KIF11 had worse survival outcomes relative to those with low KIF11. Gene set enrichment analysis (GSEA) revealed that KIF11 correlated intensively with the mevalonate (MVA) metabolic pathway. Positive associations were observed between KIF11 and MVA‐signature (HMGCR, FDFT1, SQLE, and MSMO1). KIF11 could elevate the free cholesterol content of PDAC cells and targeting MVA inhibited the in vitro growth of KIF11‐overexpressing cells. Mechanistically, we found KIF11 could interact with SREBP2, the master regulator of MVA. High KIF11 could increase SREBP2 proteins, but not alter their mRNA levels. KIF11 could attenuate the ubiquitination‐mediated degradation of SREBP2, thereby enhancing its stability and accumulation. Accordingly, KIF11 stimulated the expressions of MVA‐signature and free cholesterol contents depending on SREBP2. In addition, KIF11 depended on SREBP2 to promote cell growth, migration, stemness, and colony formation abilities. The subcutaneous xenograft models indicated that targeting MVA biogenesis (atorvastatin) is effective to restrict the in vivo growth of KIF11^high^ PDAC. Taken together, our study identified that KIF11 could activate the MVA cross talk to drive PDAC progression and inhibiting the KIF11/MVA axis provided a therapeutic vulnerability in the treatment of PDAC.

## Graphical abstract

Our study identified that KIF11 could activate the MVA crosstalk to drive PDAC progression and inhibiting KIF11/MVA axis provided a therapeutic vulnerability in the treatment of PDAC.

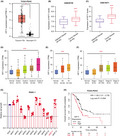

## 1 INTRODUCTION

Pancreatic cancer is a well‐known malignant disease that tends to be the second most common cause of tumor‐associated deaths worldwide.^1^, ^2^, ^3^ According to the latest statistics, the overall morbidity and mortality of pancreatic cancer are increasing annually in the United States.^4^ Pancreatic ductal adenocarcinoma (PDAC) ranks the most common type among all cases, accounting for >95% of deaths caused by pancreatic cancer.^5^ Unfortunately, a large proportion (>80%) of PDAC patients suffered from locally advanced or metastatic stages, at the time of diagnosis, thus surgical intervention with curative intent is impossible.^6^, ^7^ Although the combined strategies of radical resection and adjuvant chemotherapy developed rapidly, the overall 5‐year‐survival rate still remains low (<20%).^6^, ^8^, ^9^ As a result, novel therapeutic methodologies are urgently warranted to improve the overall prognosis of PDAC patients. In recent years, high‐throughput screening of PDAC implicated four major tumor‐driving mutations, like KRAS and TP53, of which approximately 90% of PDAC samples harbored KRAS mutations.^3^, ^10^ Collectively, our study aims to identify novel molecular vulnerabilities and subgroups of PDAC that may benefit from individual therapy to elevate the overall efficacy of treatment.

The kinesin family has 14 subfamily members classified by the motor domains that exert the roles of regulating cell mitosis and cytokinesis.^11^ Recently, owing to the functional investigations of kinesins in tumor progression and metastasis, a list of useful drugs that target microtubule dynamics were proved to be effective for clinical utility such as taxanes, epothilones, and vinca alkaloids.^12^ Nevertheless, given that the microtubule‐based cytoskeleton is not only required for tumor cell mitosis but is also essential for normal biological functions, such as sperm motility, axonal transport, or maintenance of cell polarity, the side effects of these drugs would be thorny and obvious with a dose‐dependent manner in clinical treatment.^13^, ^14^ How to screen and identify cancer‐specific kinesins for PDAC treatment is meaningful to figure out. Previous studies have already found that elevated KIF11, one member of the kinesins essential for the configuration of the bipolar spindle, correlates with poor prognosis in various malignancies, including breast cancer, lung adenocarcinoma, and meningioma.^15^, ^16^, ^17^ Monica Venere et al. also found that KIF11 expressed highly in glioblastoma to drive cell proliferation and migration, and KIF11 inhibitor could remarkably suppress initiation and self‐renewal of cancer stem cells.^18^ It is also gratifying that no apparent side effects, like neurotoxicity, were found with KIF11 inhibitors (AZD4877 and ispinesib) in the common treatment of solid tumors. However, little was known between the aberrant KIF11 levels and PDAC tumorigenesis.

As is well documented, cancer cells are experts in rewiring many metabolic cross talk to facilitate their unlimited survival and migration.^19^ Moreover, they also depend on intensive metabolic interactions with normal cells and immune cells to alter the metabolic contents in the tumor microenvironment.^20^ Intensive studies have already demonstrated that abnormal metabolic crosstalk could enhance pancreatic tumorigenesis via epigenetic regulation, implicating the essential roles of metabolism in PDAC development. In addition, many researchers have indicated that pancreatic tumor metabolism is tightly related to chemoresistance, radioresistance, or immunosuppression.^21^ Intriguingly, pancreatic cancer could also be classified into different representative subgroups with various metabolic features, including quiescent, glycolytic, cholesterogenic, and mixed groups.^22^ As a result, the metabolic characteristics of PDAC could provide significant therapeutic targets for novel and personalized treatments. Cholesterol, an essential biological component, constitutes cell membranes, which is also a precursor for steroid hormone biosynthesis.^23^ Enhanced rates of cholesterol and lipid synthesis are regarded to be an important feature of the metabolic rewiring that accelerates during the processes of cancerous transformation. Although previous documents linking the increased cholesterol intake with PDAC risk remain indefinite, results from in vivo or in vitro experimental models exhibit a causal association between them.

In the current study, we screened and revealed the biological roles of KIF11 in PDAC and found the underlying mechanisms between elevated KIF11 levels and the mevalonate (MVA) metabolism pathway. We proposed that KIF11/MVA axis might be a novel predictive biomarker and therapeutic vulnerability for PDAC progression and treatment.

## 2 MATERIALS AND METHODS

### 2.1 Cell lines and collection of patient samples

The human pancreatic cancer cell lines of SW1990, PANC‐1, and CFPAC‐1 were obtained from the Cell Bank of the Chinese Academy of Sciences (Shanghai, China). These cells were maintained in a DMEM or an RPMI‐1640 medium that contains 10% FBS, respectively. Besides, 40 patient samples were collected from the Jiangdu People’s Hospital Affiliated to Medical College of Yangzhou University. This study has been approved by the Ethics Review Committee of the Jiangdu People’s Hospital Affiliated to Medical College of Yangzhou University. All patients have signed the informed consent.

### 2.2 Colony formation assay

Cells were seeded into six‐well plates with the intensity of 1 × 10^3^/well. The medium was substituted after 72 h and cells were cultured for additional 10 days. Next, using the solution of 80% crystal violet and 20% of ethanol, cells were fixed and stained. Colonies were photographed from independent assays. Then, 30% acetic acid was added to induce a complete dissolution of the crystal violet. We detected the absorbance was at 595 nm.

### 2.3 Establishment of stable KIF11‐deficient cells via *CRISPR/Cas9* method

The pX459 plasmid was selected to clone guide oligos that target KIF11 in PDAC cells (SW1990 and PANC‐1). The SW1990 and PANC‐1 cells were plated into the 10 cm dish and then transfected with the modified pX459 plasmids for 24 h, individually. Next, 1 μg/ml puromycin was utilized to screen cells for 3 days. Then, the left living cells were seeded in 96‐well plates via limited dilution to have the monoclonal cell line. The KIF11 knockout cell clones are finally confirmed by the western blot. The sgRNA sequences that target KIF11 were listed: sgKIF11#1:F: 5′‐CACCGCACCTAATGAAGAGTATACC‐3′, sgKIF11#1:R: 5′‐AAACGGTATACTCTTCATTAGGTGC‐3′. sgKIF11#2:F: 5′‐CACCGCTTTACAGGTATGGCCAAAC‐3′, sgKIF11#1:R:5′‐AAACGTTTGGCCATACCTGTAAAGC‐3′.

### 2.4 Sphere formation assay

The SW1990 and PANC‐1 cells were collected and suspended into single cells in a nonserum medium. Then, 200 μl of nonserum medium with 200 cells/well were added into a 96‐well plate after accurate cell counting, and then each group was in 10 wells. Graphs of five randomly identified regions of each group were captured with a fluorescence microscope. The sphere percentage was determined with the number of spheres/200.

### 2.5 Western blot

After the cells were lysed in the RIPA lysis buffer. Then, the enriched cell proteins were separated on the 8% SDS‐PAGE and shifted onto PVDF membranes. We obtained the diluted primary antibodies with a concentration of 1:2000 from the Abcam company (Cambridge, MA). Next, the antibodies were used overnight after sealing in 5% skimmed milk. Then, followed by three washes in TBST, the membranes were probed with the diluted secondary antibodies with the concentration of 1:5000 for 2 h. Last, we utilized the ECL detection system to detect signals as instructed (Pierce).

### 2.6 In vitro ubiquitination assay

We extracted cell lysates from SW1990 and PANC‐1 cells on a 100‐mm cell culture plate in the immunoprecipitation (IP) buffer (Sigma). We incubated anti‐SREBP2 (ab112046) with lysate at 4°C for 16 h. We utilized the protein A/G PLUS agarose (Santa Cruz) to pull down immunocomplexes. The ubiquitinated form of SREBP2 was determined by western blot through the anti‐HA antibody.

### 2.7 Bioinformatic analysis

The mRNA levels of KIF11 were obtained from the TCGA‐PAAD cohort (https://portal.gdc.cancer.gov/), GSE28735, and GSE15471. Besides, differential analysis of KIF11 levels was conducted via the *limma* package. Besides, the Kruskal–Wallis (K–W) test was utilized to determine the associations between KIF11 levels and clinical characteristics. Kaplan–Meier analysis was conducted to assess the prognostic significance of KIF11 via the *survival* package. GSEA was conducted to enrich data sets involved in cholesterol metabolism from the MSigDB database. We reranked the genes by analyzing their RNA‐seq levels and further ranked them via “Differ_of_classes” in the GSEA software (threshold <0.05).

### 2.8 Animal assay

Four‐week‐old male nude mice from the Institute of Zoology (Beijing, China) were chosen to perform the tumor xenografts, which were further divided into two groups (*N* = 5/group). For the xenograft tumor analysis, the 1 × 10^6^ transfected SW1990 cells were injected subcutaneously into the axillary regions of mice for about 35 days. The mice were sacrificed at Day 35, and the tumor volumes or weights were detected and compared at 1–4 weeks. All animal experiments were approved by the Animal Care Committee of the Jiangdu People’s Hospital Affiliated to Medical College of Yangzhou University.

### 2.9 Statistical analysis

The Student’s *t* test was selected to determine differences between two groups, whereas the log‐rank test was utilized to analyze the differences in survival. All experimental data were indicated as mean ± standard deviation (SD). Statistical analysis was conducted using the GraphPad Prism 7.0 software. A value of *p* < 0.05 was considered statistically significant.

## 3 RESULTS

### 3.1 High KIF11 correlates with poor prognosis and shorter overall survival (OS)

**FIGURE 1:**
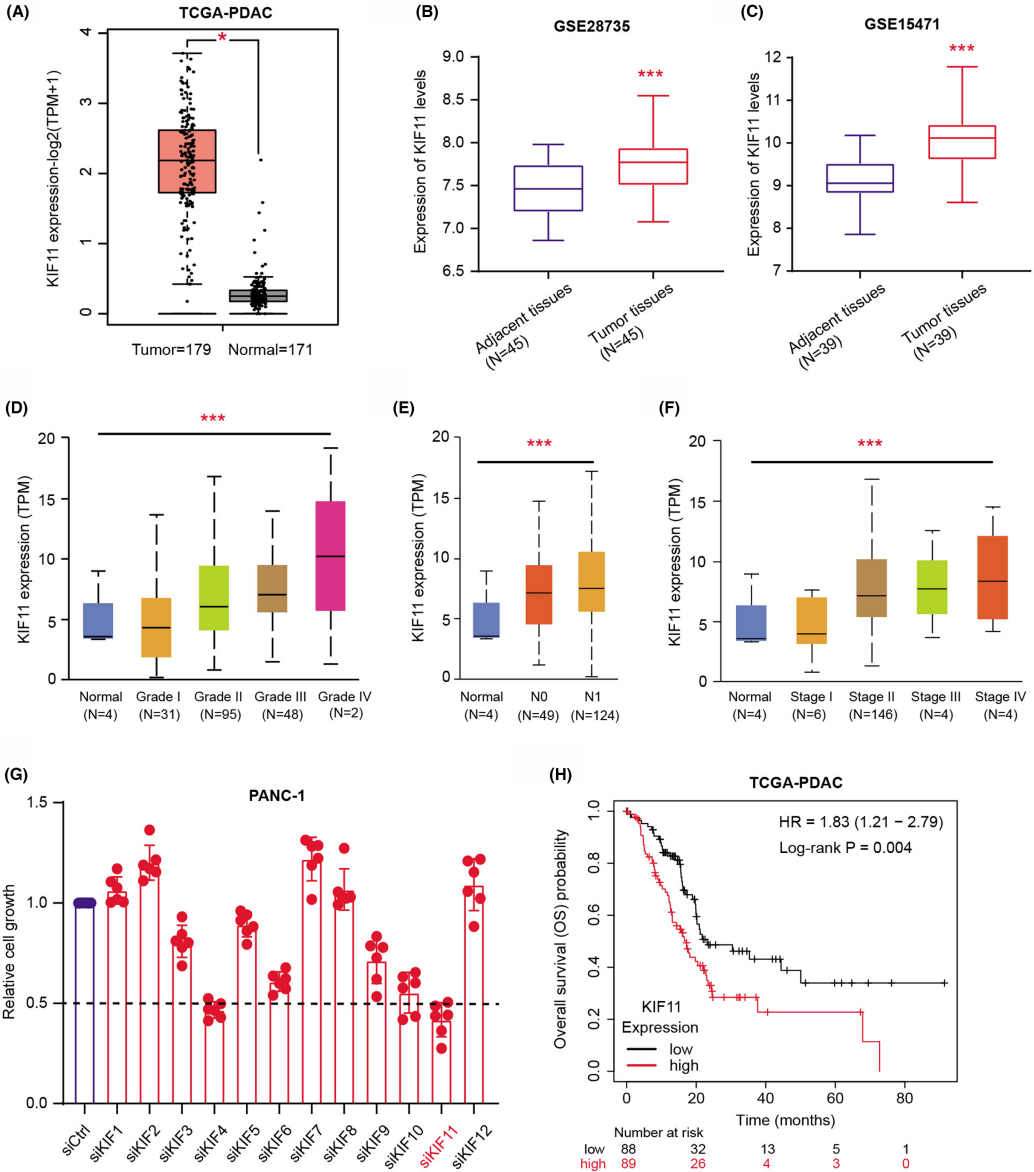
High KIF11 was a hazard factor in PDAC that correlated with poor prognosis and shorter overall survival. (A) Differential analysis of KIF11 levels between tumor and normal samples in TCGA‐PRAD cohort. (B‐C) Differential analysis of KIF11 mRNA levels between adjacent tissues and tumor tissues in GSE28735 data set (B) and GSE15471 data set (C). (D–F) Correlation analysis based on TCGA‐PRAD samples revealed the positive associations among KIF11 levels and tumor grades (D), lymphatic stages, (E) and clinicopathological stages (F). (G) MTT assays reveal the cell growth in cells transfected with siRNAs targeting each kinesin family member, individually. (H) Kaplan–Meier analysis comparing the differences of survival outcomes between KIF11‐high and KIF11‐low PDAC samples. *p < 0.05, **p < 0.01, ***p < 0.001

To explore the clinical significance of KIF11 in PDAC, we first downloaded the mRNA levels of KIF11 from 179 patients in the TCGA‐PDAC cohort (https://portal.gdc.cancer.gov/). Besides, expression data of KIF11 in normal pancreatic tissues were also collected from the GTEx data set (https://www.gtexportal.org/home/index.html). The differential analysis was conducted to find that KIF11 mRNA levels were notably upregulated in tumor samples relative to those in normal tissues with *p* < 0.001 (Figure 1A). We also validated the high expression levels of KIF11 in PDAC in other two independent data set, including GSE28735 (*N* = 45, *p* < 0.001, Figure 1B) and GSE15471 (*N* = 39, *p* < 0.001, Figure 1C). Correlation analysis further confirmed that high KIF11 expressions were positively associated with tumor grades (*p* < 0.001, Figure 1D), lymphatic metastasis (*p* < 0.001, Figure 1E), and clinicopathological stages (*p* < 0.001, Figure 1F). Intensive studies have already indicated the potential associations between kinesin family members with tumor progression. We thus selected several members and designed specific siRNAs to conduct the MTT assays. Relative to most of the other kinesin members, KIF11 inhibition induced the most remarkable decrease in PANC‐1 cell growth (Figure 1G). Last, we also collected the clinical characteristics of PDAC samples from the TGCA cohort and matched the survival data with expression data of KIF11. PDAC patients were accordingly categorized into KIF11^high^ and KIF11^low^ groups to conduct the Kaplan–Meier analysis, in which patients with high KIF11 had worse overall survival (OS) outcomes with shorter time compared with those with low KIF11 levels (N = 177, log‐rank test *p* < 0.001, Figure 1H). Collectively, we concluded that KIF11 is a hazard factor in PDAC and has vital clinical significance.

### 3.2 Validation of high KIF11 levels in PDAC samples

**FIGURE 2:**
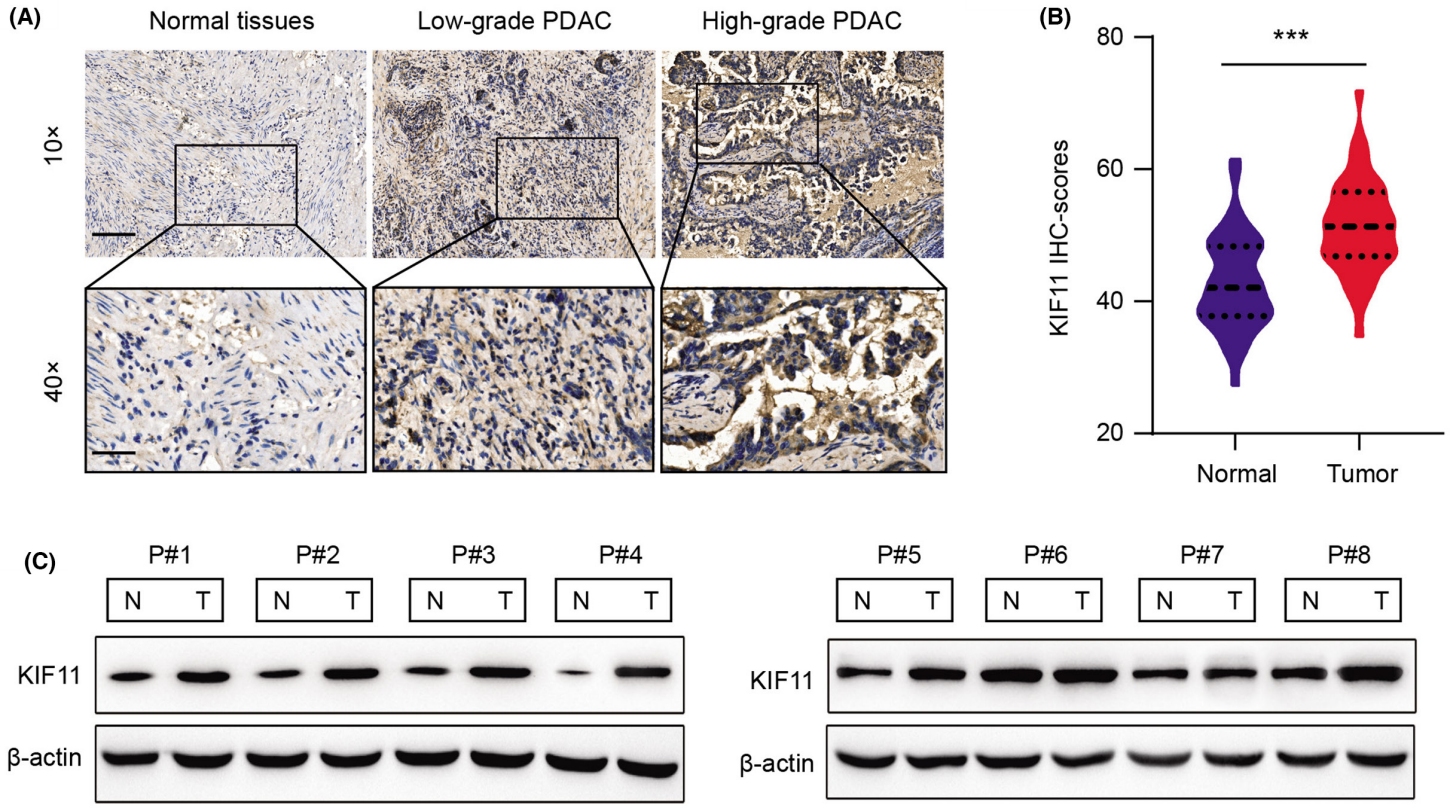
Validation of KIF11 levels in resected PDAC samples. (A) Representative IHC staining graph indicated low and high KIF11 levels in paired normal tissues and tumor samples with different grades. Upper graph (scale bar = ), Lower graph (scale bar = ). (B) Quantification of KIF11 staining levels in normal and tumor samples. C Western blotting assay showing the protein levels of KIF11 in tumor and normal fresh PDAC tissues (*N* = 8). *p < 0.05, **p < 0.01, ***p < 0.001

Given that these findings were observed in public data sets, we also collected totally 40 pairs of PDAC samples matched with normal tissues from the department of radiotherapy in our hospital. The immunohistochemistry (IHC) staining showed that KIF11 expression levels were not only high in tumors versus normal samples but correlated with high tumor grades (Figure 2A). Besides, differential analysis of KIF11 levels (H scores) further confirmed these results (Figure 2B). Consistent with these observations, the protein levels of KIF11 were also found to be notably higher at 7/10 (70%) human PDAC tumors than in their paired normal pancreatic tissues via western blot assays (Figure 2C). Taken together, our study validated that KIF11 was higher in tumor samples versus normal tissues, in line with the previous results.

### 3.3 KIF11 promotes cell proliferation, migration, and self‐renewal abilities in PDAC

**FIGURE 3:**
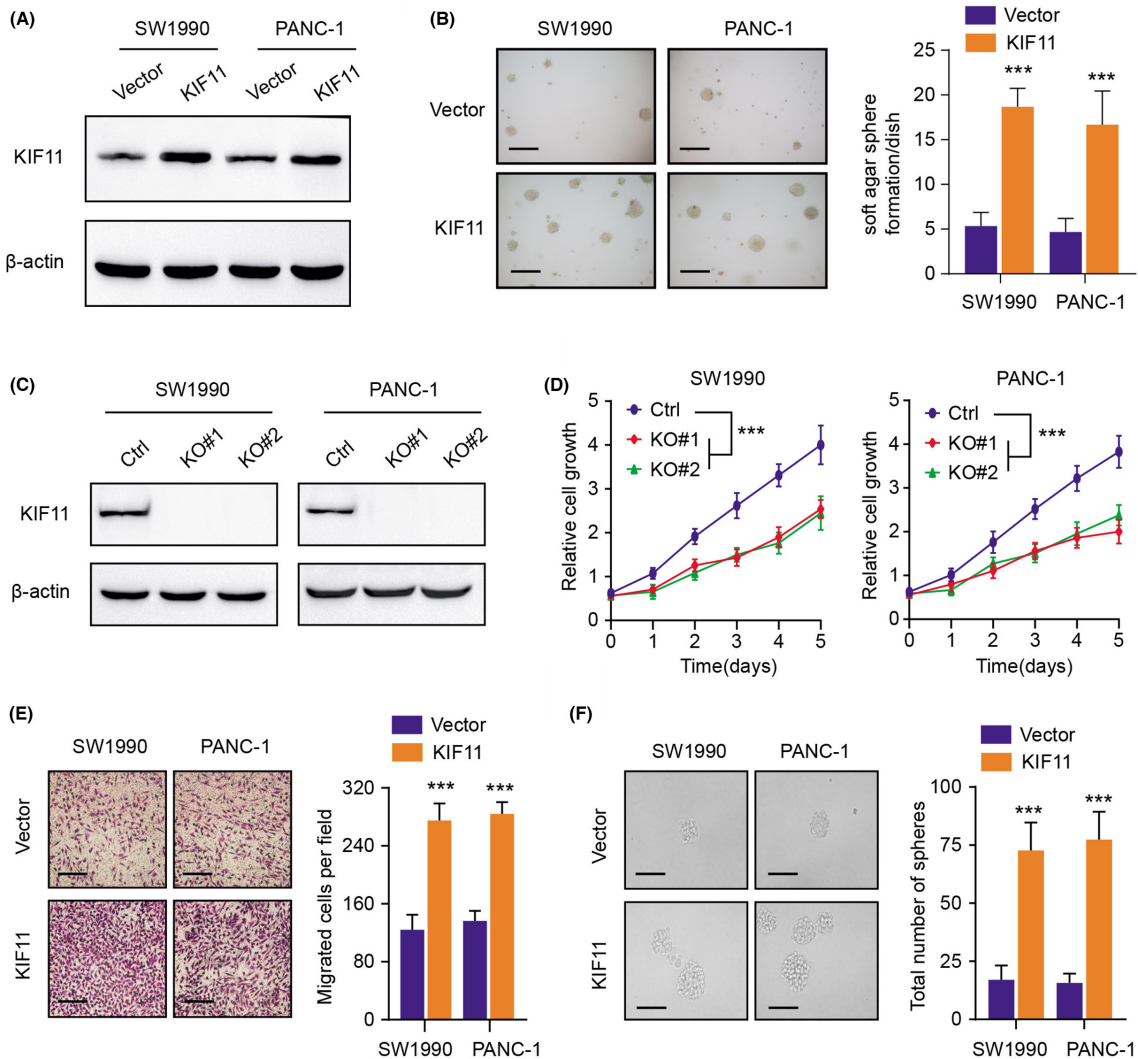
KIF11 overexpression enhanced PDAC cells’ malignant features. (A) Detection of KIF11 protein levels in cells (SW1990 and PANC‐1) transfected with vector and KIF11 via western blotting assay. (B) Upregulation of KIF11 promoted the anchorage‐independent growth abilities of PDAC cells (SW1990 and PANC‐1) (scale bars = 250 μm, left panel). Quantification of the soft agar assay results was shown in the right panel. (C) Western blotting assay revealing the protein levels of KIF11 in WT cells and KIF11‐KO cells (SW1990 and PANC‐1). (D) Cell growth analysis via CCK‐8 assays of PDAC cells (SW1990 and PANC‐1) with or without KIF11 depletion (*CRISPR/Cas9*‐mediated KO). (E) Cell migration assays of PDAC cells (SW1990 and PANC‐1) transfected with vector and KIF11. Representative pictures (scale bars = 250 μm, left panel) and quantification of results were shown (right panel). (F) Sphere formation assays in cells transfected with vector and KIF11. Representative graphs (left panel, scale bars = 250 μm) and quantification of results were shown (right panel). *p < 0.05, **p < 0.01, ***p < 0.001

To assess the function of KIF11 in PDAC, we first constructed stable KIF11‐overexpressing (KIF11‐OE) PDAC cells (SW1990 and PANC‐1) and detected the protein levels via western blot (Figure 3A). The PDAC cell (SW1990 and PANC‐1) soft agar colony formation efficiency was apparently enhanced in KIF11‐overexpressing cells relative to cells transfected with vector (Figure 3B). Besides, we also utilized the *CRISPR/Cas9* technology to delete the KIF11 gene and confirmed the knockout efficiency via western blot and Sanger sequencing (Figure 3C). KIF11 depletion could also suppress the soft agar colony formation efficiency of PDAC cells (PANC‐1, SW1990) in Figure S1A. CCK‐8 assays revealed that KIF11 deficiency could significantly reduce the growth capacity of SW1990 and PANC‐1 cells compared with control WT cells, whereas KIF11 overexpression could enhance cell viability (Figure 3D and Figure S1B). Furthermore, KIF11 overexpression could reinforce the migration ability of cells, as quantified by the transwell assays (Figure 3E). Last, the self‐renewal ability of PDAC cells was also elevated with KIF11 overexpression, indicating that KIF11 could promote stemness features of tumors (Figure 3F). Taken together, our study suggested that KIF11 may act as an oncogene that enhances PDAC proliferation, migration, and stemness.

### 3.4 High KIF11 promotes PDAC progression through modulating the mevalonate (MVA) metabolism pathway

**FIGURE 4:**
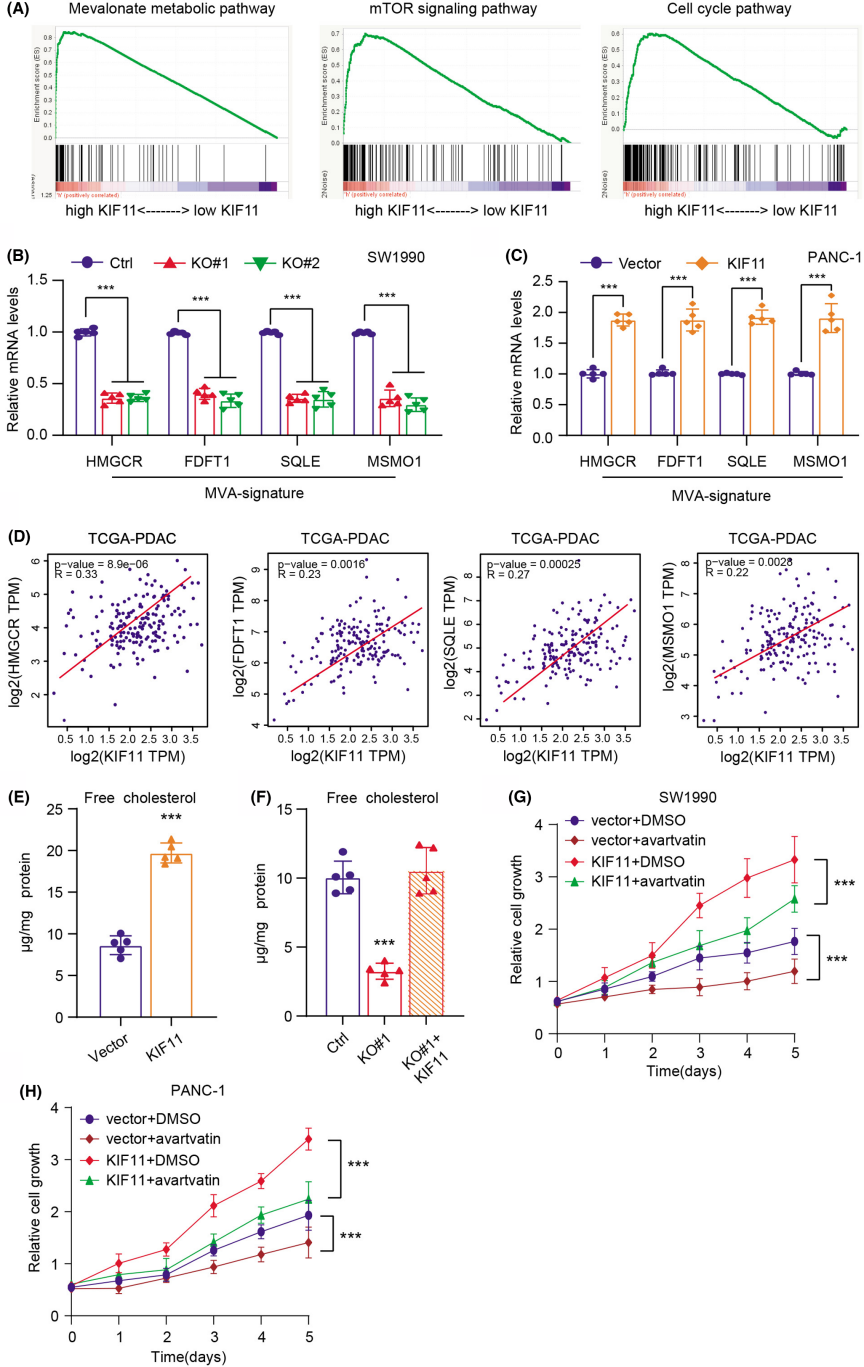
KIF11 promoted PDAC progression via activating the mevalonate (MVA) metabolism pathway. (A) Gene Set Enrichment Analysis (GSEA) was performed in TCGA‐PRAD samples, and several oncogenic cross talk was highly enriched in KIF11‐high samples, including the mevalonate pathway. (B) The levels of MVA‐signature (HMGCR, FDFT1, SQLE, and MSMO1) expressions were detected in WT and KIF11‐depleted cells by qRT‐PCR. (C) The levels of MVA‐signature (HMGCR, FDFT1, SQLE, and MSMO1) expressions were detected in cells transfected with vector and KIF11 via qRT‐PCR. (D) Correlation analysis was conducted among KIF11 and MVA‐signature (HMGCR, FDFT1, SQLE, and MSMO1) in TCGA‐PRAD samples. (E) Quantification of free cholesterol contents in cells transfected with vector and KIF11. (F) Quantification of free cholesterol contents in three cell groups (Ctrl, KO#1, and KO#1 + KIF11). (G) MTT analysis in three SW1990 cell groups (Vector + DMSO, Vector + atorvastatin, KIF11 + DMSO, KIF11 + atorvastatin). (H) MTT analysis in three PANC‐1 cell groups (Vector + DMSO, Vector + atorvastatin, KIF11 + DMSO, KIF11 + atorvastatin). *p < 0.05, **p < 0.01, ***p < 0.001

To further identify the molecular mechanisms by which KIF11 promotes PDAC progression, we thus conducted the Gene Set Enrichment Analysis (GSEA) via analyzing the expression data of the TCGA‐PDAC cohort (Figure 4A). Given that the cell cycle is a common pathway involved in tumorigenesis of multiple tumors. Meanwhile, cholesterol metabolism could stimulate mTORC1 activation, highlighting that cholesterol could accelerate tumorigenesis of PDAC. As a result, we focused on the MVA metabolism of PDAC for further investigation. Intriguingly, we observed that KIF11 expressions and the mevalonate metabolic pathway were highly interrelated in pancreatic cancer from TCGA. Besides, we detected the specific genes via qRT‐PCR analysis and found that essential MVA pathway genes, such as HMGCR, FDFT1, SQLE, and MSMO1, were all decreased in KIF11‐deficient cells versus parental control cells (Figure 4B). However, the mRNA levels of HMGCR, FDFT1, SQLE, and MSMO1 were all consistently elevated in KIF11‐OE cells relative to cells transfected with vector (Figure 4C). We further found that KIF11 correlated significantly with the MVA pathway signature based on the TCGA‐PDAC samples (Figure 4D). In line with the findings, the free cholesterol content in KIF11‐OE cells was about 40% higher than in controls (Figure 4E). KIF11 deficiency indeed reduced the free cholesterol content, but an ectopic expression of KIF11 could restore the levels of cholesterol detected in the cell culture medium (Figure 4F). Last, suppression of cholesterol synthesis (atorvastatin) could remarkably inhibit the KIF11‐OE cell growth compared with DMSO, indicating that mevalonate (MVA) metabolism is indispensable for KIF11‐driven tumorigenesis (Figure 4G‐H). Taken together, our study revealed that KIF11 could manipulate the mevalonate (MVA) metabolism to promote PDAC growth.

### 3.5 KIF11 interacts with SREBP2 and stabilizes its proteins from ubiquitination‐mediated degradation

**FIGURE 5:**
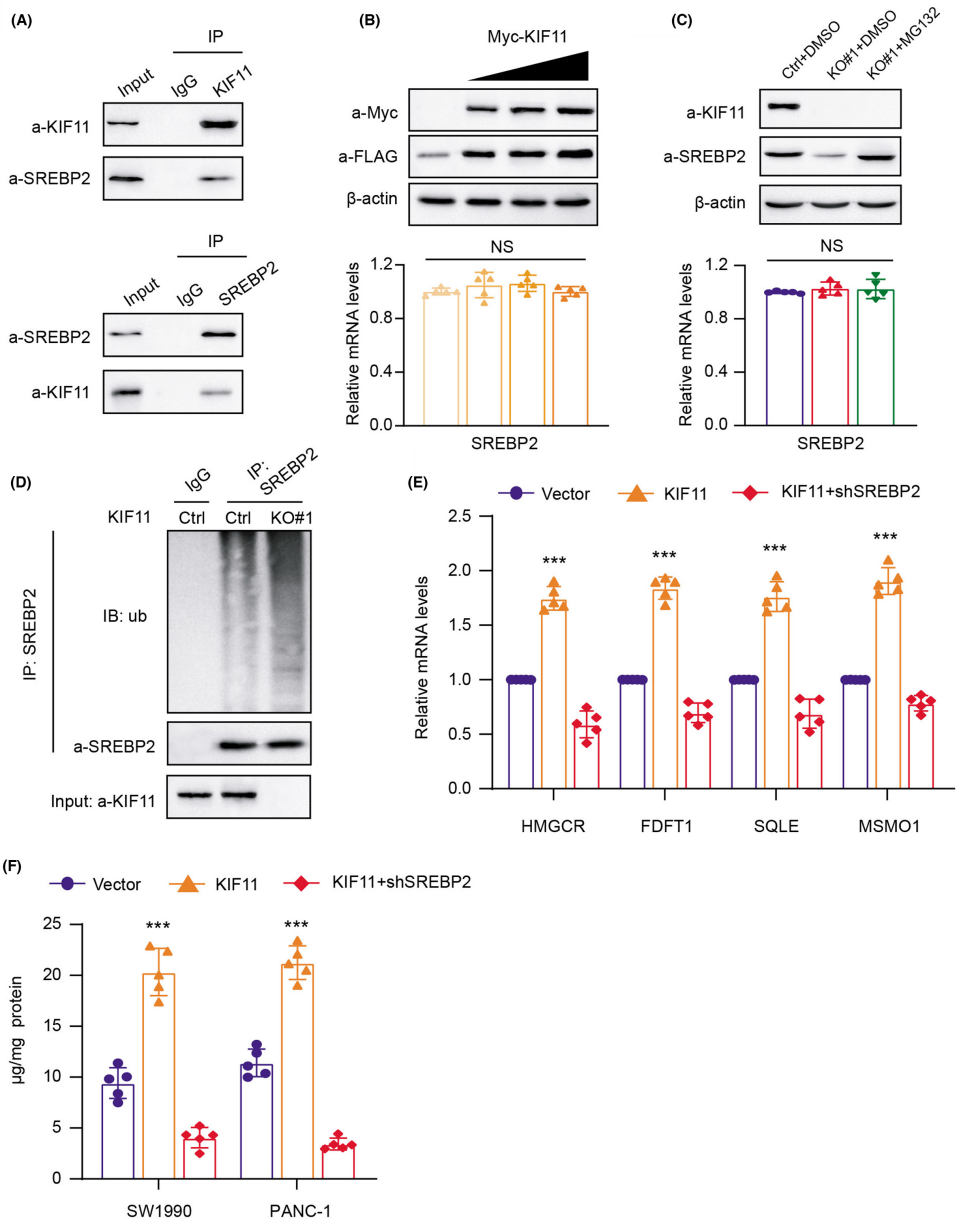
KIF11 interacts with SREBP2 and stabilizes its proteins from ubiquitination‐mediated degradation. (A) Western blot of indicated proteins in WCLs and Co‐IP samples of IgG or anti‐KIF11 antibody obtained from the cell extracts of SW1990 cells treated with 10 μM of MG132 for 10 h (upper panel). This result was confirmed by reciprocal Co‐IP assay with anti‐SREBP2 antibody (lower panel). (B) Western blotting assay detecting the SREBP2 proteins in cells transfected with increasing amounts of Myc‐KIF11 plasmids (upper panel). Quantification of Flag‐SREBP2 mRNA levels via qPCR was shown on the lower panel. (C) Western blot assay detecting the endogenous SREBP2 proteins in three cell groups (Ctrl + DMSO, KO#1 + DMSO, and KO#1 + MG132) (upper panel). Quantification of corresponding SREBP2 mRNA levels was shown on the lower panel. (D) Western blot detecting the products of in vivo SREBP2 ubiquitination assays from the indicated SW1990 cells treated with 30 μM MG132 for 8 h. (E) Quantification of mRNA expression levels of MVA‐signature via qPCR in three cell groups (Vector, KIF11, and KIF11 + shSREBP2). (F) Detection of free cholesterol contents in PDAC cells (Vector, KIF11, and KIF11 + shSREBP2). *p < 0.05, **p < 0.01, ***p < 0.001

Given that nearly most of the essential enzymes of sterol biosynthetic pathways are modulated by SREBP2, we thus wondered whether KIF11 could correlate with dysregulation of SREBP2. First of all, we conducted the Co‐IP assays using the anti‐KIF11 antibody and anti‐SREBP2 antibody in PANC‐1 cell lysate, individually. KIF11 could successfully immunoprecipitate SREBP2 (Figure 5A). Reciprocally, SREBP2 was able to immunoprecipitate KIF11 effectively, suggesting the endogenous interactions between the two proteins (Figure 5A). Also, we thus cotransfected the PANC‐1 cells with Flag‐SREBP2 and Myc‐KIF11 and found that SREBP2 proteins steadily increased when the amount of KIF11 is elevating (Figure 5B). However, no changes in mRNA levels of SREBP2 were observed under this condition (Figure 5B). Besides, KIF11 deficiency indeed resulted in the decrease of SREBP2 proteins, but not mRNA levels, which could be completely restored with the treatment of MG132, one well‐known proteasome inhibitor (Figure 5C). Briefly, we considered that KIF11 could stabilize the SREBP2 in a dose‐dependent manner at the posttranscriptional levels. In line with our above speculations, we conducted the in vitro ubiquitination assay and observed that KIF11 deficiency could lead to an apparent increase of robust polyubiquitination of SREBP2 (Figure 5D). Last, we observed that SREBP2 ablation could largely abrogate the effect of KIF11‐OE on transcription of mevalonate (MVA)‐signature, indicating that KIF11 depends on SREBP2 to drive activation of the MVA pathway (Figure 5E). KIF11 could activate the free cholesterol concentrations in PDAC cells in an SREBP2‐dependent manner, which could be further abolished by SREBP2 ablation (Figure 5F). Taken together, our study indicated that KIF11 stabilizes SREBP2 to modulate the mevalonate (MVA) crosstalk.

### 3.6 Inhibition of MVA biogenesis (atorvastatin) is effective to suppress KIF11^high^ PDAC

**FIGURE 6:**
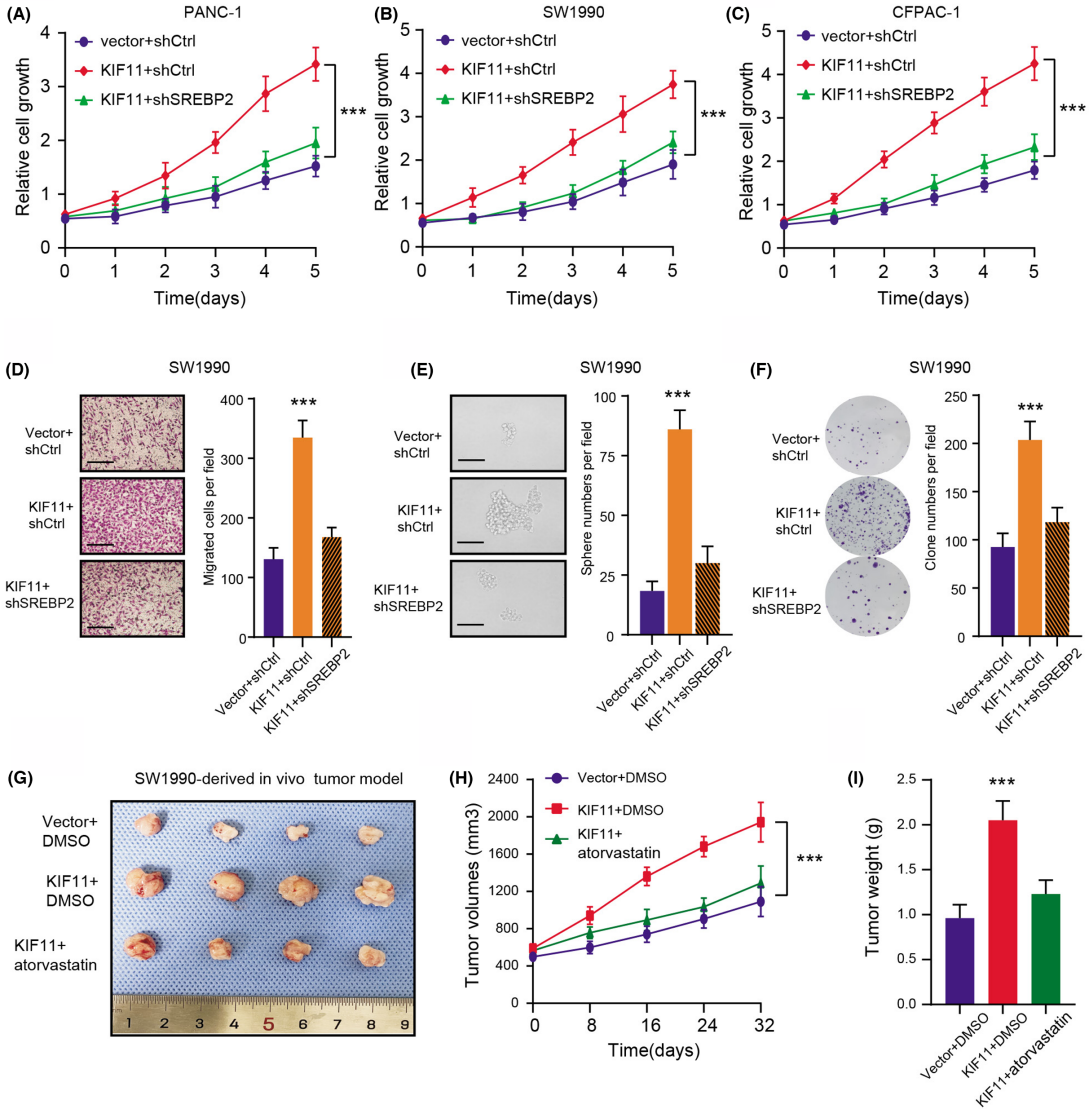
Targeting MVA biogenesis (atorvastatin) is effective to inhibit the growth of KIF11^high^ PDAC. (A‐C) MTT analysis detecting the cell growth of three groups (Vector + shCtrl, KIF11 + shCtrl, and KIF11 + shSREBP2) in PANC‐1 (A), SW1990 (B) and CFPAC‐1 (C), respectively (C). (D) Representative pictures (scale bars = 250 μm) were shown on the left panel. Quantification results of the cell migration abilities of KIF11‐overexpressing SW1990 cells transfected with the SREBP2 shRNAs or their corresponding controls were shown on the right panel. (E) Representative graphs showing the self‐renewal potentialities of KIF11‐overexpressing SW1990 cells with or without shSREBP2 (left panel, scale bars = 250 μm). Quantification results were shown on the right. (F) Representative graphs (left panel) and quantification data (right panel) of the colony formation abilities of KIF11‐overexpressing SW1990 cells transfected with the SREBP2 shRNAs or their corresponding controls. (G) The SREBP2 knockdown suppressed KIF11‐induced SW1990 PDAC cells subcutaneous tumor growth of nude mice. (H) Tumor volumes in three groups (Vector + DMSO, KIF11 + DMSO, KIF11 + atorvastatin) were measured and compared. (I) Tumor weight was monitored and compared in three groups (Vector + DMSO, KIF11 + DMSO, KIF11 + atorvastatin). *p < 0.05, **p < 0.01, ***p < 0.001

Given that the SREBP2‐driven MVA pathway is indispensable for the progression of PDAC, we thus speculated that targeting SREBP2 could be effective to suppress KIF11‐OE PDAC. First of all, we found that SREBP2 knockdown could largely reduce the cell growth induced by KIF11‐OE as indicated by the CCK‐8 assays (Figure 6A–C). Besides, we also observed that KIF11‐OE enhanced cell migration ability, which could be largely impaired with SREBP2 knockdown (Figure 6D). In addition, we also conducted the sphere formation assay, where KIF11‐driven tumor stemness features could be notably suppressed with SREBP2 knockdown (Figure 6E). Last, we further constructed the subcutaneous xenograft models and found that atorvastatin was proved to be effective to suppress KIF11‐OE PDAC progression, as quantified by tumor volumes and tumor weights (Figure 6F–H). Collectively, our findings indicated that targeting the MVA pathway (atorvastatin) is a useful strategy to inhibit KIF11^high^ PDAC tumor growth.

## 4 DISCUSSION

Tumor cells may remodel the essential metabolism to supply essential contents and energy that are required for maintaining aberrant growth, which frequently appears via either mutation in key enzymes, like isocitrate dehydrogenases (IDHs), or alterations in cell signals induced by abnormal nutrients and tumor microenvironment.^24^, ^25^ Meanwhile, these abnormal signaling cascades could further disturb the expression or activity of enzymes in vital metabolic cross talk, including the mevalonate (MVA) pathway.^26^ The MVA pathway utilizes acetyl‐CoA, NADPH, and ATP to produce sterols and isoprenoids that are vital for tumor proliferation. Following glucose, glutamine, or acetate consumption, the production of acetyl‐CoA accelerates in cancer cells. Besides, the NADPH is derived from multiple processes, including malic enzymes, IDHs, and the pentose phosphate pathway.^27^, ^28^ As a result, the MVA pathway is intensively integrated into the whole metabolic pathways in cancer cells. The MVA pathway has been indicated by multiple researches to be oncogenic. Sung‐Hwan Moon et al. found that p53 could block the activation of SREBP‐2, the key transcriptional manipulator of the MVA pathway, via transcriptionally inducing the ABCA1 cholesterol transporter gene.^29^ In another study, mevalonate inhibition in cancer cells disrupts Rac1 prenylation to enhance recognition and cross‐presentation by conventional dendritic cells, providing a potential target for cancer immunotherapy. In addition, the MVA was found to induce an escape mechanism of tumor growth in HER2^+^ breast cancer models that are resistant to anti‐HER2 drugs. MVA inhibitors could be useful therapeutic agents to resensitize the tumors to anti‐HER2 therapies.^30^ Collectively, these evidence indicate that the MVA pathway has an essential role in cancer.

In the current study, we performed the MTT assay to screen that targeting KIF11 induced the sharp decrease of PDAC cell growth relative to other members of the kinesin family. Differential analysis suggested that KIF11 expressed highly in tumor cases compared with those in normal tissues, and the results were consistently confirmed in multiple PDAC data sets and resected fresh PDAC samples. Besides, we found that KIF11 levels correlate with advanced stages and patients with high KIF11 levels had a poor prognosis, indicating that KIF11 is a meaningful biomarker for PDAC prediction. We further investigated the functional roles of KIF11 in PDAC and KIF11 overexpression significantly promoted tumor cell growth, migration, and self‐renewal potentialities. However, KIF11 ablation by *CRISPR/Cas9*‐mediated knockout could notably suppress cell growth. GSEA analysis indicated the casual link between KIF11 and the mevalonate metabolic pathway, and KIF11 promoted the expressions of MVA‐associated genes. Accordingly, KIF11 could elevate the cellular free cholesterol levels and depended on the MVA pathway to enhance cell growth. Intriguingly, KIF11 could mediate the stability of SREBP‐2 proteins to drive the transcriptional levels of downstream targets. Last, we also demonstrated that KIF11 replied on SREBP2 to promote cell growth, colony formation, or migration abilities and maintain stemness features. Targeting the SREBP2‐driven MVA pathway (atorvastatin) could significantly suppress KIF11‐induced in vivo tumor growth.

Previous efforts have been made to elucidate the underlying mechanisms that contribute to aberrant activation of MVA cross talk.^31^, ^32^ Recently, ZMYND8 was proved to interact with SREBP2 to coordinate enhancer–promoter interaction to stimulate the MVA cross talk.^33^ The SREBP2/ZMYND8 associates with the mediator complex to promote the transcriptional activities of MVA‐associated genes, such as HMGCR, SQLE, FDFT1, or FDPS. Besides, loss of p53 promotes murine liver tumorigenesis and correlates with increased SREBP‐2 maturation, indicating that the mevalonate pathway plays a crucial role in the p53‐mediated liver tumor suppression.^29^ Furthermore, MIEF2 (mitochondrial elongation factor 2) increased SREBP1 and SREBP2 by activating ROS/AKT/mTOR signaling to drive the lipid synthesis in ovarian cancer cells.^34^ In this study, we found that KIF11 could hardly regulate the mRNA levels of SREBP2 and mainly impacts the posttranslational (PTM) modification of SREPB2, which has been little reported. KIF11 could interact with SREBP2 and protect SREBP2 from ubiquitination‐mediated degradation. As a result, KIF11 overexpression could stabilize the cellular SREBP2 to drive expressions of its downstream signature, which could be completely abolished with SREBP2 knockdown, implicating that KIF11 regulates MVA cross talk in an SREBP2‐dependent manner. Previous studies have suggested that an aberrant ubiquitin‐proteasome system (UPS) could participate in the disorders of cholesterol pathways. The liver X receptor LXR inhibits the low‐density lipoprotein (LDL) uptake via transcriptional induction of Idol (inducible degrader of the LDLR), thereby targeting it for degradation.^35^ Moreover, corylin treatment could partially inhibit the Akt activity at the Thr308 site, specifically enhancing mSREBPs ubiquitination and proteasomal degradation to restrict the lipid content in liver cell lines.^36^ Collectively, in line with previous findings, we linked the KIF11 levels with activation of the MVA pathway in PDAC through aberrant ubiquitination‐dependent degradation of SREBP2.

Currently, the clinical efficacy of statins, the well‐known inhibitor of cholesterol biogenesis mevalonate (MVA) pathway, is overall indefinite, mainly due to a lack of patient classification criteria.^36^, ^37^ In this study, we found atorvastatin could be effective to inhibit cell growth and migration. Besides, atorvastatin could largely suppress the in vivo tumor growth in KIF11^high^ PDAC cells. As a result, we proposed that specific MVA pathway inhibitors could be useful to treat PDAC, especially for KIF11^high^ samples. However, more preclinical mouse models or patient‐derived tumor xenografts (PDXs) are warranted to evaluate the differential drug responses of atorvastatin in KIF11^high^ and KIF11^low^ PDAC samples.

Nevertheless, we still found several defects that need to be further improved in the current study. First of all, large PDAC samples were warranted to assess the clinical significance and prognostic significance of KIF11 in patients. Second, we could utilize the immunohistochemistry (IHC) to score the KIF11 staining levels and identify the optimal threshold to stratify KIF11^high^ and KIF11^low^ patients. Given that KIF11 depended on SREBP2 to drive PDAC progression, the differential antitumor effects between KIF11 inhibitors and atorvastatin were not well elucidated and compared. Last of all, although we have explored that KIF11 could drive PDAC progression, migration, and stemness abilities, the underlying associations between KIF11 and immune regulations in the PDAC microenvironment still remain inconclusive, which is meaningful for further investigations in the following studies.

## 5 CONCLUSION

In summary, we identified that high KIF11 is an oncogenic factor in PDAC. Mechanistically, KIF11 stabilizes SREBP2 proteins from ubiquitination‐mediated degradation. Besides, KIF11 depended on elevated SREBP2 proteins to drive MVA crosstalk, and targeting the MVA pathway is effective to suppress tumor growth. Therefore, KIF11/SREBP2 axis may function as a potential predictor and therapeutic vulnerability for PDAC.

## ETHICAL APPROVAL STATEMENT

The human tissue specimens and clinical data were reviewed and approved by the Jiangdu People’s Hospital Affiliated to Medical College of Yangzhou University (Jiangsu, China). All patients have signed the written informed consent permitted by the Ethics Review Committee of the Jiangdu People’s Hospital Affiliated to Medical College of Yangzhou University.

## CONFLICT OF INTEREST

The authors declare that the research was conducted in the absence of any commercial or financial relationships that could be construed as a potential conflict of interest.

## AUTHOR CONTRIBUTIONS

Songbing Qin conceived the concept of this study. Xiang Gu and Qunshan Zhu conducted the experimental assays. Guangyu Tian, Wenbo Song, and Tao Wang collected the samples from patients. Xiang Gu, Ali Wang, and Xiaojun Chen performed the statistical analysis. Xiang Gu wrote the paper and Songbing Qin conducted the revisions. All authors have approved the final draft of the paper.

## Supporting information


Figure S1

Table S1

## Data Availability

The molecular experiment data generated and analyzed during the current study are available from the corresponding author on reasonable request.

## References

[1] Mizrahi JD , Surana R , Valle JW , Shroff RT . Pancreatic cancer. Lancet. 2020;395(10242):2008‐2020. 32593337 10.1016/S0140-6736(20)30974-0

[2] Ho WJ , Jaffee EM , Zheng L . The tumour microenvironment in pancreatic cancer ‐ clinical challenges and opportunities. Nat Rev Clin Oncol. 2020;17(9):527‐540. 32398706 10.1038/s41571-020-0363-5 PMC7442729

[3] Buscail L , Bournet B , Cordelier P . Role of oncogenic KRAS in the diagnosis, prognosis and treatment of pancreatic cancer. Nat Rev Gastroenterol Hepatol. 2020;17(3):153‐168. 32005945 10.1038/s41575-019-0245-4

[4] Siegel RL , Miller KD , Fuchs HE , Jemal A . Cancer statistics, 2021. CA Cancer J Clin. 2021;71(1):7‐33. 33433946 10.3322/caac.21654

[5] Puleo F , Nicolle R , Blum Y , et al. Stratification of pancreatic ductal adenocarcinomas based on tumor and microenvironment features. Gastroenterology. 2018;155(6):1999‐2013.e3. 30165049 10.1053/j.gastro.2018.08.033

[6] Grossberg AJ , Chu LC , Deig CR , et al. Multidisciplinary standards of care and recent progress in pancreatic ductal adenocarcinoma. CA Cancer J Clin. 2020;70(5):375‐403. 32683683 10.3322/caac.21626 PMC7722002

[7] Tian C , Öhlund D , Rickelt S , et al. Cancer cell‐derived Matrisome proteins promote metastasis in pancreatic ductal adenocarcinoma. Cancer Res. 2020;80(7):1461‐1474. 32029550 10.1158/0008-5472.CAN-19-2578 PMC7127978

[8] Bengtsson A , Andersson R , Ansari D . The actual 5‐year survivors of pancreatic ductal adenocarcinoma based on real‐world data. Sci Rep. 2020;10(1):16425. 33009477 10.1038/s41598-020-73525-y PMC7532215

[9] McGuigan A , Kelly P , Turkington RC , Jones C , Coleman HG , McCain RS . Pancreatic cancer: a review of clinical diagnosis, epidemiology, treatment and outcomes. World J Gastroenterol. 2018;24(43):4846‐4861. 30487695 10.3748/wjg.v24.i43.4846 PMC6250924

[10] Li JT , Yin M , Wang D , et al. BCAT2‐mediated BCAA catabolism is critical for development of pancreatic ductal adenocarcinoma. Nat Cell Biol. 2020;22(2):167‐174. 32029896 10.1038/s41556-019-0455-6

[11] Loncar A , Rincon SA , Lera Ramirez M , Paoletti A , Tran PT . Kinesin‐14 family proteins and microtubule dynamics define *S. pombe* mitotic and meiotic spindle assembly, and elongation. J Cell Sci. 2020;133(11):jcs240234. 32327557 10.1242/jcs.240234 PMC7295595

[12] Prota AE , Bargsten K , Zurwerra D , et al. Molecular mechanism of action of microtubule‐stabilizing anticancer agents. Science. 2013;339(6119):587‐590. 23287720 10.1126/science.1230582

[13] Zajączkowska R , Kocot‐Kępska M , Leppert W , Wrzosek A , Mika J , Wordliczek J . Mechanisms of chemotherapy‐induced peripheral neuropathy. Int J Mol Sci. 2019;20(6):1451. 10.3390/ijms20061451 PMC6471666 30909387

[14] Vahdat LT , Garcia AA , Vogel C , et al. Eribulin mesylate versus ixabepilone in patients with metastatic breast cancer: a randomized phase II study comparing the incidence of peripheral neuropathy. Breast Cancer Res Treat. 2013;140(2):341‐351. 23877339 10.1007/s10549-013-2574-2 PMC3732762

[15] Zhou J , Chen WR , Yang LC , et al. KIF11 functions as an oncogene and is associated with poor outcomes from breast cancer. Cancer Res Treat. 2019;51(3):1207‐1221. 30590004 10.4143/crt.2018.460 PMC6639218

[16] Li Z , Yu B , Qi F , Li F . KIF11 serves as an independent prognostic factor and therapeutic target for patients with lung adenocarcinoma. Front Oncol. 2021;11:670218. 33968780 10.3389/fonc.2021.670218 PMC8103954

[17] Jungwirth G , Yu T , Cao J , et al. KIF11 inhibitors filanesib and ispinesib inhibit meningioma growth in vitro and in vivo. Cancer Lett. 2021;506:1‐10. 33652084 10.1016/j.canlet.2021.02.016

[18] Venere M , Horbinski C , Crish JF , et al. The mitotic kinesin KIF11 is a driver of invasion, proliferation, and self‐renewal in glioblastoma. Sci Transl Med. 2015;7(304):304ra143. 10.1126/scitranslmed.aac6762 PMC4743764 26355032

[19] Dias AS , Almeida CR , Helguero LA , Duarte IF . Metabolic crosstalk in the breast cancer microenvironment. Eur J Cancer. 2019;121:154‐171. 31581056 10.1016/j.ejca.2019.09.002

[20] Pavlova NN , Thompson CB . The emerging hallmarks of cancer metabolism. Cell Metab. 2016;23(1):27‐47. 26771115 10.1016/j.cmet.2015.12.006 PMC4715268

[21] Wang T , Fahrmann JF , Lee H , et al. JAK/STAT3‐regulated fatty acid β‐oxidation is critical for breast cancer stem cell self‐renewal and chemoresistance. Cell Metab. 2018;27(1):136‐150.e5. 29249690 10.1016/j.cmet.2017.11.001 PMC5777338

[22] Son J , Lyssiotis CA , Ying H , et al. Glutamine supports pancreatic cancer growth through a KRAS‐regulated metabolic pathway. Nature. 2013;496(7443):101‐105. 23535601 10.1038/nature12040 PMC3656466

[23] Huang B , Song BL , Xu C . Cholesterol metabolism in cancer: mechanisms and therapeutic opportunities. Nat Metab. 2020;2(2):132‐141. 32694690 10.1038/s42255-020-0174-0

[24] Qing Y , Dong L , Gao L , et al. R‐2‐hydroxyglutarate attenuates aerobic glycolysis in leukemia by targeting the FTO/m(6)a/PFKP/LDHB axis. Mol Cell. 2021;81(5):922‐939.e9. 33434505 10.1016/j.molcel.2020.12.026 PMC7935770

[25] Han S , Liu Y , Cai SJ , et al. IDH mutation in glioma: molecular mechanisms and potential therapeutic targets. Br J Cancer. 2020;122(11):1580‐1589. 32291392 10.1038/s41416-020-0814-x PMC7250901

[26] Mullen PJ , Yu R , Longo J , Archer MC , Penn LZ . The interplay between cell signalling and the mevalonate pathway in cancer. Nat Rev Cancer. 2016;16(11):718‐731. 27562463 10.1038/nrc.2016.76

[27] Clark O , Yen K , Mellinghoff IK . Molecular pathways: isocitrate dehydrogenase mutations in cancer. Clin Cancer Res. 2016;22(8):1837‐1842. 26819452 10.1158/1078-0432.CCR-13-1333 PMC4834266

[28] Pramono AA , Rather GM , Herman H , Lestari K , Bertino JR . NAD‐ and NADPH‐contributing enzymes as therapeutic targets in cancer: an overview. Biomolecules. 2020;10(3):358. 10.3390/biom10030358 PMC7175141 32111066

[29] Moon SH , Huang CH , Houlihan SL , et al. p53 represses the mevalonate pathway to mediate tumor suppression. Cell. 2019;176(3):564‐80.e19. 30580964 10.1016/j.cell.2018.11.011 PMC6483089

[30] Sethunath V , Hu H , De Angelis C , et al. Targeting the mevalonate pathway to overcome acquired anti‐HER2 treatment resistance in breast cancer. Mol Cancer Res. 2019;17(11):2318‐2330. 31420371 10.1158/1541-7786.MCR-19-0756 PMC6825570

[31] Göbel A , Zinna VM , Dell'Endice S , et al. Anti‐tumor effects of mevalonate pathway inhibition in ovarian cancer. BMC Cancer. 2020;20(1):703. 32727400 10.1186/s12885-020-07164-x PMC7388525

[32] Chen CL , Paul LN , Mermoud JC , Steussy CN , Stauffacher CV . Visualizing the enzyme mechanism of mevalonate diphosphate decarboxylase. Nat Commun. 2020;11(1):3969. 32769976 10.1038/s41467-020-17733-0 PMC7414129

[33] Pan Q , Zhong S , Wang H , et al. The ZMYND8‐regulated mevalonate pathway endows YAP‐high intestinal cancer with metabolic vulnerability. Mol Cell. 2021;81(13):2736‐51.e8. 33932349 10.1016/j.molcel.2021.04.009

[34] Zhao S , Cheng L , Shi Y , Li J , Yun Q , Yang H . MIEF2 reprograms lipid metabolism to drive progression of ovarian cancer through ROS/AKT/mTOR signaling pathway. Cell Death Dis. 2021;12(1):18. 33414447 10.1038/s41419-020-03336-6 PMC7791105

[35] Zelcer N , Hong C , Boyadjian R , Tontonoz P . LXR regulates cholesterol uptake through idol‐dependent ubiquitination of the LDL receptor. Science. 2009;325(5936):100–4. 19520913 10.1126/science.1168974 PMC2777523

[36] Zheng ZG , Zhang X , Liu XX , et al. Inhibition of HSP90β improves lipid disorders by promoting mature SREBPs degradation via the ubiquitin‐proteasome system. Theranostics. 2019;9(20):5769‐5783. 31534518 10.7150/thno.36505 PMC6735373

[37] Laraia L , Friese A , Corkery DP , et al. The cholesterol transfer protein GRAMD1A regulates autophagosome biogenesis. Nat Chem Biol. 2019;15(7):710‐720. 31222192 10.1038/s41589-019-0307-5

