# Supplementary figures and images for: KIF11 manipulates SREBP2‐dependent mevalonate cross talk to promote tumor progression in pancreatic ductal adenocarcinoma
Source: Cancer Med. 2022 May 26;11(17):3282–95. doi: 10.1002/cam4.4683 (PMC9468433; doi:10.1002/cam4.4683)

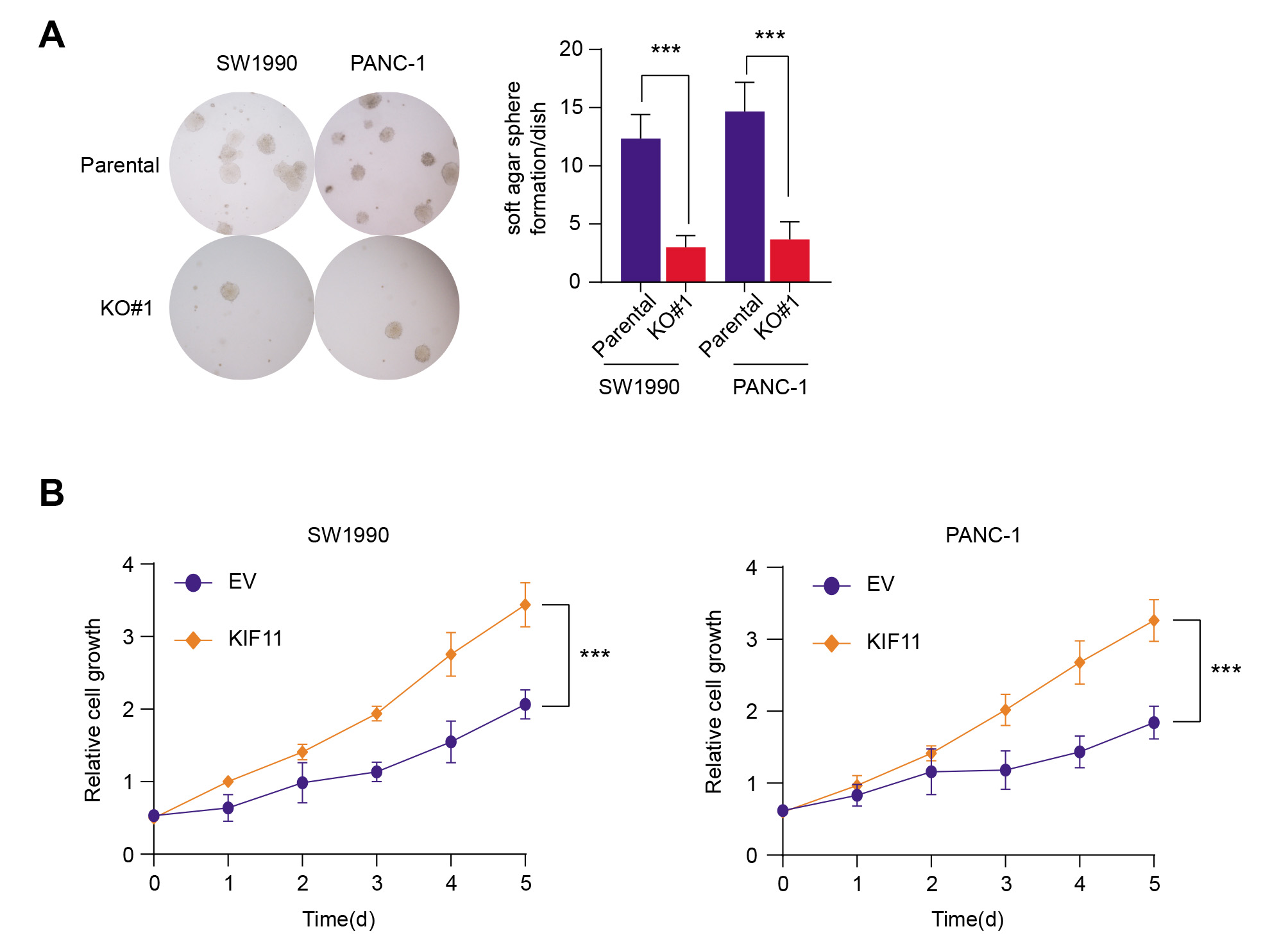

Supplement: Supplementary file 1 — Figure S1 [file CAM4-11-3282-s002.jpg]
